# Low peak expiratory flow and subsequent first self-report of recognized lung disease: Person-disjoint replication and spirometric benchmarking in HRS and ELSA

**DOI:** 10.1371/journal.pone.0360668

**Published:** 2026-10-12

**Authors:** Haiyang Hu, Chun Wang, Jiang Fu, Haining Zhou

**Affiliations:** 1 Department of Thoracic Surgery, Suining Central Hospital, Suining, Sichuan, China; 2 Institute of Surgery, Graduate School, Chengdu University of Traditional Chinese Medicine, Chengdu, China; Marwadi University, INDIA

## Abstract

**Background:**

Low peak expiratory flow (PEF) may precede the first self-report of recognized lung disease, but cross-cohort consistency and incremental value are uncertain.

**Methods:**

We analysed Health and Retirement Study (HRS) discovery and prespecified person-disjoint replication samples and the English Longitudinal Study of Ageing (ELSA). The estimand linked baseline PEF to the first subsequently observed self-report of recognized lung disease, not biological onset. HRS used a prespecified nominal lower-decile z score and ELSA an empirical lower decile. Participant-clustered models adjusted for demographic, clinical and functional factors. Weighting and a stroke comparator were sensitivity analyses. Post-hoc ELSA benchmarking compared PEF with FEV1 and FVC using person-disjoint cross-validation and full-pipeline bootstrap sensitivity.

**Results:**

Models included 18,510 HRS discovery windows (320 reports), 11,424 replication windows (188), and 8,634 ELSA person-waves (95). Low PEF was associated with first report in discovery (odds ratio [OR] 3.36, 95% confidence interval [CI] 2.54–4.45), replication (3.14, 2.19–4.49), and ELSA (3.77, 2.36–6.01). Weighted estimates were similar; stroke associations were weaker or null. After adjustment for separate FEV1 and FVC indices, the continuous-PEF OR was 1.04 (0.90–1.20). In 1,584 fixed-horizon participants (95 events), AUCs were 0.650 for covariates, 0.684 with PEF, 0.745 with FEV1 and FVC, and 0.739 with all three. Full-pipeline bootstrap sensitivity found no clear incremental evidence beyond FEV1 and FVC. Across differing horizons, low-PEF rules identified fewer than one-third of reports in HRS replication and ELSA.

**Conclusions:**

Lower PEF was associated with a subsequent first observed lung-disease report; cohort-specific low-PEF rules identified fewer than one-third. In exploratory fixed out-of-fold predictions, PEF improved average precision and prediction error over covariates; no clear incremental evidence remained after FEV1 and FVC. Same-protocol collection does not establish a peak-flow-only pathway.

## Introduction

Chronic respiratory disease is often recognized late, and a recorded diagnosis does not always agree with post-bronchodilator spirometry. Population studies have documented unrecognized airflow obstruction in older adults, while primary-care evidence shows both underdiagnosis and overdiagnosis of chronic obstructive pulmonary disease (COPD) [1–3]. Reviews confirm that both forms of diagnostic error remain common across settings [4]. A recent randomized trial also showed that active case finding can identify previously undiagnosed COPD or asthma and increase treatment, underscoring the clinical importance of the interval between physiological impairment and disease recognition [5]. That interval is shaped not only by disease progression but also by symptoms, healthcare contact, access to testing, diagnostic labelling and patient recall.

Symptoms alone may not bring people to respiratory assessment. In community and primary-care studies, targeted approaches identified obstructive disease that routine care had missed, although the yield and downstream uptake varied [6–8]. This is distinct from screening asymptomatic adults, which is not recommended because available evidence shows no net benefit [9]. A low-cost physiological signal could therefore be useful as a prompt for targeted assessment without being suitable as a population screening or diagnostic test.

Peak expiratory flow (PEF) is quick to measure and is available in several longitudinal studies of ageing. Low PEF has been associated with later disability, healthcare use and mortality [10,11]. However, PEF depends on expiratory effort, technique and respiratory muscle function [12,13] and has also been used to operationalize respiratory sarcopenia in older adults [14]; it cannot establish airflow obstruction. Systematic reviews and validation studies indicate that flow-based measures can contribute to COPD case-finding, but generally perform best alongside symptoms, exposure history or other lung-function tests [15–19]. COPD diagnosis still requires quality-assured post-bronchodilator spirometry [20].

Broader pre-COPD frameworks distinguish early structural or functional abnormalities from established COPD [21]. Longitudinal studies have also examined PEF before respiratory outcomes. Low PEF was associated with a later self-reported physician diagnosis of chronic lung disease in the China Health and Retirement Longitudinal Study [22]. Subsequent work related repeated PEF-muscle trajectories to incident chronic disease [23]. Other Chinese cohorts have examined low PEF as a pre-COPD indicator and in relation to later FEV1/FVC decline [24,25]. These studies support temporal association, but leave two practical questions: whether the association persists when repeat participants are separated between discovery and a prespecified replication, and whether PEF adds useful predictive information when FEV1 and FVC are already available.

We addressed these questions in two national ageing cohorts. HRS provided discovery and prespecified person-disjoint replication samples, and ELSA provided an independent cohort with a different PEF scale and follow-up structure. The primary estimand was the adjusted association between baseline PEF and the first subsequently observed self-report of recognized lung disease among records with observed outcome status; it was not biological incidence or a causal effect. Secondary analyses examined weighting, incident stroke as an imperfect non-respiratory comparator, absolute risk, and case-finding performance. An exploratory post-hoc ELSA analysis then benchmarked PEF against separately released FEV1 and FVC indices. Because these measurements were collected during the same nurse assessment, the comparison concerns the information carried by the recorded indices rather than substitution of a separate peak-flow device for spirometry.

## Materials and methods

### Study design and data sources

This longitudinal study used HRS for discovery and prespecified person-disjoint replication and ELSA for an independent cohort analysis. HRS follows a nationally representative sample of US adults older than 50 years and their spouses, usually at two-year intervals [26]. ELSA is a multidisciplinary panel of adults aged 50 years or older living in England, with biennial interviews and periodic nurse assessments [27]. HRS analyses used the RAND HRS Longitudinal File 1992–2022 version 1 and the 2022 tracker file (https://hrsdata.isr.umich.edu/data-products/rand). ELSA data came from UK Data Service Study 5050 (https://doi.org/10.5255/UKDA-SN-5050-37) and comprised the wave-6 nurse assessment and harmonized waves 6–10 interview data. Reporting follows the STROBE statement [28].

The primary estimand was the adjusted association between baseline PEF and the first subsequently observed self-report of recognized lung disease among HRS risk-set windows or ELSA person-waves with observed outcome status. It did not estimate biological disease onset, disease incidence independent of observation, or a causal effect of PEF.

HRS even-wave physical-measure panels formed the discovery sample. HRS odd-wave panels formed the person-disjoint replication sample; participants who met the discovery-contributor definition were removed before replication models were fitted. Before that analysis, we fixed eligibility, the two replication windows, PEF construction and threshold, outcome definition, covariate sets, model form, and planned sensitivity analyses. Regression coefficients, standard errors, confidence intervals, and predictions were re-estimated in the replication sample; no fitted discovery equation or prediction was transported unchanged. The ELSA analysis was specified after related ELSA spirometric results had been inspected and was not preregistered. Survey weighting, the stroke comparator, adjustment for separately released FEV1 and FVC indices, and post-event PEF were sensitivity or exploratory analyses. Repeated-cross-validation benchmarking of the ELSA lung-function indices was added after the principal association analyses and is reported as post hoc.

### Ethical statement

The parent HRS protocols were approved by the University of Michigan Institutional Review Board, and HRS obtained participant consent. ELSA wave 6 was approved by the National Research Ethics Service Committee South Central-Berkshire (11/SC/0374); ELSA documents the approvals for later waves and obtains participant consent (https://www.elsa-project.ac.uk/ethical-approval). This secondary analysis used de-identified data under the parent studies’ data-use conditions and involved no new participant recruitment or contact. For this study, the HRS data were accessed for research purposes on 11 May 2026, and the ELSA data were accessed on 14 May 2026. The authors did not have access to information that could identify individual participants during or after data collection. The authors’ institutions did not conduct an additional ethics review.

### HRS analytic panels

The HRS discovery analysis used three observation triplets: waves 8-9-10, 10-11-12, and 12-13-14. PEF and covariates were taken from the first wave in each triplet, the first subsequent lung-disease report was assessed at the middle wave, and PEF at the third wave was reserved for the exploratory post-event analysis. Person-disjoint replication used waves 9-10-11 and 11-12-13. Participants were eligible for a lung-disease window when they reported no prior lung disease at baseline. A participant could contribute more than one eligible window, so all regression standard errors were clustered by person.

The prespecified replication rule excluded every person who had an observed baseline PEF, no baseline lung-disease report, and an observed subsequent lung-disease outcome in any discovery window. There were 9,679 discovery contributors and 8,148 candidate replication contributors with usable PEF and an observed outcome before exclusion. Two participants met both definitions and were removed. The broader at-risk replication pool after this exclusion contained 18,748 windows from 12,446 participants; 8,146 participants had usable PEF and an observed outcome.

### ELSA analytic sample

The ELSA baseline was the wave-6 nurse spirometry assessment. The cohort-analysis risk set was restricted to participants aged 60–95 years who were recorded as White, had grade A or B spirometry, valid positive FEV1, FVC, PEF, and height measurements, no baseline respiratory-disease report, no recorded lung or asthma prescription, and no recent inhaler use. Among 2,849 eligible participants, 2,670 had at least one observed lung-disease follow-up at waves 7–10 and contributed 8,634 person-wave records; 179 had no observed follow-up and did not enter the person-wave association analysis. Follow-up records were censored at the first report or the last eligible observation.

### Lung-function measurement

HRS lung function was measured during the enhanced face-to-face physical-measure visit. The physical-measures protocol is documented by HRS (https://hrs.isr.umich.edu/sites/default/files/biblio/dr-011.pdf). Interviewers used a Mini-Wright Peak Flow Meter with a disposable mouthpiece and sought three readings 30 seconds apart; participants were asked to stand, inhale deeply and blow as hard and fast as possible, with a seated position permitted when necessary. The RAND wave-level variable records the maximum non-missing value from the three measurements in L/min. Beginning in 2006, physical measures were offered to a randomly selected half-sample; participants interviewed by proxy, in nursing homes or by telephone did not complete them.

ELSA wave-6 lung function was measured by pre-bronchodilator nurse spirometry with an ndd Easy On-PC spirometer. The public health-visit protocol is available from UK Data Service (https://doc.ukdataservice.ac.uk/doc/5050/mrdoc/pdf/5050_elsa_waves_2_4_6_8_9_11_health_visit_data_user_guide.pdf). The protocol sought three acceptable blows and allowed three to eight attempts. The released HTPEF, HTFEV, and HTFVC variables contain the separately highest technically satisfactory PEF, FEV1, and FVC values; PCPEF contains the instrument-provided percent-predicted PEF. Because these maxima could come from different blows and the public file did not contain manoeuvre-level records or a verifiable device-generated FEV1/FVC value, FEV1 and FVC were entered separately and no ratio was reconstructed. Participants could receive a copy of their results and, with separate consent, results were generally sent to their general practitioner within approximately three months. The archived analytic file did not include the GP-release consent indicator. Baseline abnormalities may therefore have increased diagnostic attention, and the observed association cannot separate latent respiratory impairment from a measurement-to-recognition pathway.

### PEF exposures

In HRS, PEF values outside 1–900 L/min were treated as invalid. Within each wave and sex, PEF was regressed on linear and quadratic terms for age and height and an indicator for a sitting measurement position. Residuals were divided by their model residual standard deviation to create a within-wave adjusted PEF z score. The continuous contrast was expressed per 1-SD lower adjusted PEF. Operational low PEF was fixed before replication as an adjusted residual z score below −1.2816, corresponding to the nominal lower decile under a standard normal distribution. This was not an empirical bottom decile and selected approximately 8% of the analytic HRS windows.

ELSA used the instrument-provided percent-predicted PEF value. The continuous contrast was expressed per 10 percentage-points lower percent-predicted PEF. The binary threshold was the empirical lower decile of percent-predicted PEF in the eligible wave-6 risk set, which corresponded to less than 77% predicted. The cohort-specific continuous scales were not pooled because the HRS residual and ELSA instrument-provided percentage represented different measurement constructs. Cohort-specific low-PEF contrasts were displayed side by side without treating the thresholds as identical.

### Outcomes

In HRS, eligibility required the RAND baseline indicator RwLUNGE = 0. The primary outcome was RwLUNGS = 1 at the next wave, denoting a first observed report of lung disease since the previous interview. In ELSA, participants reporting no chronic lung disease or asthma at wave 6 were followed for the first observed self-report of doctor-diagnosed chronic lung disease, such as chronic bronchitis or emphysema, at waves 7–10. A broader chronic lung disease-or-asthma indicator was a secondary outcome. These outcomes locate first observed recognition or reporting, not pathological onset or a newly adjudicated diagnosis. Incident stroke among participants without baseline stroke served as an imperfect non-respiratory comparator. Because it shares determinants with both PEF and lung-disease reporting, it could neither establish respiratory specificity nor remove residual confounding.

The exploratory HRS post-event outcome was adjusted PEF at the final wave of each triplet. It was regressed on the intervening first lung-disease report while controlling for baseline PEF and the systemic covariate set. This analysis was kept outside the main claim because its person-disjoint replication did not meet the prespecified replication criterion and because diagnosis, treatment, smoking change, healthcare contact, reporting behaviour, and test effort could all affect later PEF.

### Covariates and missing data

HRS models used a sequentially defined set of covariates. The demographic set included age, sex, race, Hispanic ethnicity, years of education, and window indicators. The clinical set added body mass index, ever and current smoking, self-rated health, an eight-item Center for Epidemiologic Studies Depression score, hospitalization, and a count of hypertension, diabetes, cancer, heart disease, stroke, and arthritis. The systemic set additionally included age-, sex-, and height-adjusted grip strength, activities of daily living, instrumental activities of daily living, and mobility limitations. The systemic model was the principal adjusted analysis. HRS regression models used complete records for the exposure, outcome, and covariates required by each model; no missing exposure or outcome was imputed. Participant flow and the additional loss at each complete-record step are reported in Fig 1 and S2 Table.

**Fig 1:**
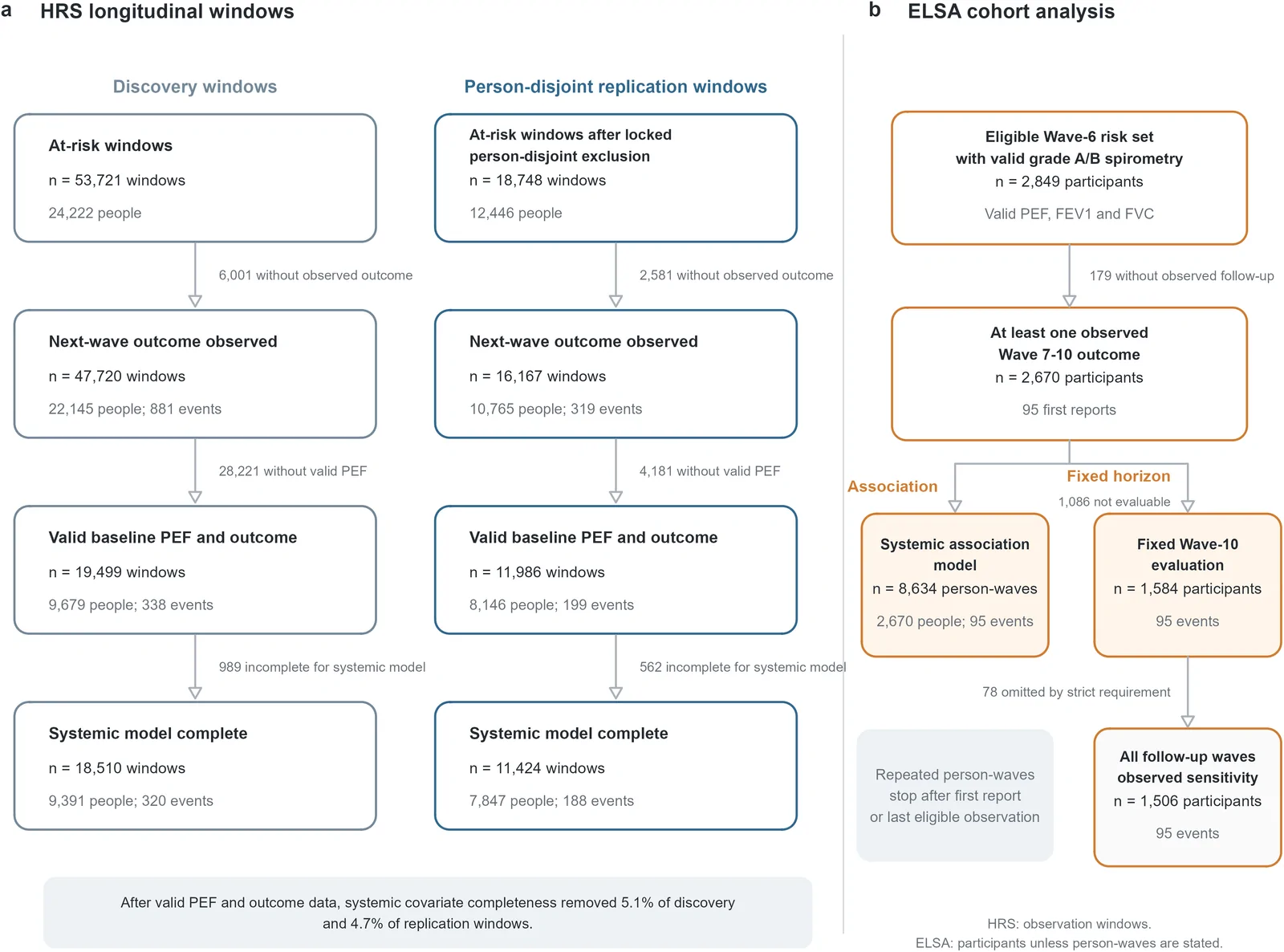
Cohort and analytic-sample flow in HRS and ELSA. a, HRS discovery and prespecified person-disjoint replication observation windows. Each sequence begins with windows in which no prior lung disease was reported and shows availability of the next-wave lung-disease outcome, a valid baseline PEF residual, and complete records for the systemic adjustment set. Counts below window totals give unique participants and, once the outcome is observed, first lung-disease reports. Participants can contribute more than one HRS window. The prespecified replication rule excluded discovery contributors; two otherwise eligible replication participants overlapped with discovery contributors. b, ELSA participants with valid grade A/B wave-6 spirometry and no baseline chronic respiratory report, followed by those with at least one observed wave 7-10 outcome. The association analysis used 8,634 person-waves from 2,670 participants. Fixed-horizon performance was evaluated in 1,584 participants whose status was classifiable under the study rule: all 95 first reports plus event-free participants with an observed wave-10 response. The strict sensitivity set required observed outcomes at every follow-up wave. Sequential loss labels are arithmetic differences between adjacent displayed stages. HRS death and other nonresponse are tabulated separately in S2 Table. PEF, peak expiratory flow; HRS, Health and Retirement Study; ELSA, English Longitudinal Study of Ageing.

ELSA demographic models included follow-up-wave indicators, age and sex. Clinical adjustment added smoking status, cigarette consumption, body mass index, education, hypertension, diabetes, heart disease, stroke where appropriate, cancer and spirometry quality. The systemic model added adjusted grip strength, activities of daily living, instrumental activities of daily living, mobility limitations, self-rated health and depressive symptoms. Missing-indicator terms accompanied prespecified replacements for cigarette consumption among current smokers, body mass index, education, grip strength, functional measures, self-rated health and depressive symptoms. Spirometric-volume-adjusted association and benchmarking models used separate FEV1 and FVC deficit z scores derived from the Global Lung Function Initiative 2012 Caucasian equations [29].

### Outcome non-observation and death

No missing exposure or outcome was imputed. In HRS, next-wave tracker status allowed deaths before outcome ascertainment to be separated descriptively from ordinary nonresponse and withdrawal. These windows contributed to the disposition analysis but not to association models, and deaths were not coded as event-free. In ELSA, the harmonized interview-status fields available in the eligible analytic extract contained no death-specific terminal codes for waves 7–10; the vital status of nonrespondents could not be separated reliably from other unknown horizon status. We therefore report this limitation rather than treating unobserved participants as event-free or estimating a cross-cohort competing-risk model.

### Statistical analysis

Associations with first disease report were estimated using repeated-window or discrete-time logistic regression. HRS models were fitted as binomial generalized linear models, and ELSA models maximized the corresponding logistic likelihood. Sandwich standard errors were clustered by participant in both cohorts. The systemic model was the principal adjusted analysis; estimates from earlier adjustment sets were not interpreted as attenuation because their complete-record samples differed. Effect estimates are reported as odds ratios (ORs) with 95% confidence intervals (CIs). Exact two-sided P values are provided.

The HRS weighting sensitivity analysis used wave-specific physical-measure respondent weights normalized to mean one within baseline wave. These weights target the baseline enhanced face-to-face physical-measure half-sample and measured differential participation in that examination; they do not weight longitudinal outcome response, recover outcomes after death or other nonresponse, or address selection related to unmeasured factors. Other analyses specified before HRS person-disjoint replication comprised window-specific estimates with a descriptive heterogeneity check, stabilized inverse-probability weighting for observation of endpoint PEF, and exclusion of baseline stroke in the non-respiratory comparator analysis. ELSA weighted association models multiplied the wave-6 nurse weight by a stabilized inverse probability of observed follow-up response estimated from follow-up wave and non-spirometric baseline covariates. Response weights were truncated at the first and 99th percentiles [30]. Crude risks and the sensitivity, specificity, positive predictive value (PPV) and negative predictive value (NPV) of the binary low-PEF definition were calculated for operational interpretation. Wilson intervals were used for plotted crude risks. Because HRS assessed next-wave reports whereas ELSA accumulated reports through wave 10, cohort-specific sensitivity, specificity, PPV, and NPV were not treated as directly comparable.

Benjamini-Hochberg q values were calculated separately within each ELSA outcome family: first chronic lung-disease report, the combined chronic lung-disease-or-asthma report, and incident stroke. Each family comprised continuous PEF in the demographic, clinical, and systemic models; binary low PEF in the systemic model; and both contrasts in the survey-plus-response-weighted systemic models. The principal respiratory associations remained supported after correction; no principal conclusion depended on choosing an unadjusted rather than adjusted P-value threshold. HRS and ELSA estimates were displayed side by side but were not pooled because exposure construction, eligibility, and follow-up differed. Main association analyses were implemented in Python 3.12.13, and figures were rendered in R 4.3.1. Analysis code, package-version information, and instructions for reproducing the analyses from authorised HRS and ELSA data are provided in S1 Code.

After the main manuscript analyses, we conducted an exploratory post-hoc ELSA spirometric-volume benchmarking analysis in R 4.3.1. The fixed wave-10 evaluation set included all 95 participants with a first observed report during follow-up and 1,489 event-free participants with an observed event-free wave-10 response (n = 1,584). Thus, the estimand was predictive performance among participants whose horizon status was classifiable under this observation rule, not among all 2,849 baseline-eligible participants. Inclusion depended partly on the observed outcome process. A strict sensitivity set required an observed outcome at every follow-up wave (n = 1,506; 95 events). Pooled discrete-time logistic models used a primary low-dimensional adjustment set of follow-up-wave indicators, age, sex, smoking status and intensity, body mass index, education, and corresponding missingness indicators; the manuscript systemic set was retained as an overfitting-sensitive secondary analysis. Within each adjustment set, four predefined variants contained covariates alone, covariates plus continuous PEF, covariates plus separate FEV1 and FVC deficit z scores, or FEV1 and FVC plus continuous PEF. No FEV1/FVC ratio or data-derived PEF cut-point was used in this benchmarking analysis.

Five-fold participant-disjoint cross-validation was repeated five times using seed 20260724, with every record from one participant kept in the same fold. Within each training fold, body mass index imputation, grip-strength residualization and residual scaling, and centering and scaling of functional measures, self-rated health and depressive symptoms were estimated from unique training participants and then applied unchanged to test participants. Person-wave probabilities were combined as cumulative risk through wave 10. Performance measures were receiver operating characteristic area under the curve (AUC), average precision, Brier score, log loss, calibration-in-the-large and calibration slope [31–34]. The originally submitted paired intervals used 2,000 participant-level bootstrap resamples of averaged out-of-fold predictions and were conditional on those predictions. In response to review, we added a 500-replicate participant bootstrap that repeated participant resampling, fold allocation, fold-local preprocessing, all four model fits, out-of-fold prediction, and performance estimation. Each bootstrap replicate used one five-fold participant-disjoint cross-validation cycle; duplicate bootstrap occurrences of an original participant remained in the same fold. Percentile intervals from this full pipeline include sampling, fold-allocation, preprocessing, model-fitting, and evaluation uncertainty. All 500 replicates completed successfully.

We assessed selection into fixed-horizon evaluation by endpoint-observation weighting within the 2,670-person longitudinal cohort. A logistic nuisance model predicted whether wave-10 endpoint status was classifiable from non-spirometric baseline covariates and explicit missingness indicators. Stabilized weights were truncated at the first and 99th percentiles, normalized to mean one, and applied identically to the four sets of averaged out-of-fold predictions. This analysis targets selection into endpoint observation, not a sequential censoring process. It cannot reconstruct intermittent nonresponse, death, and disease recognition, and death-specific status could not be identified reliably in the eligible ELSA extract. We therefore retained the fixed-horizon analysis as exploratory and gave the all-waves-observed sensitivity analysis separate prominence. Bootstrap intervals for endpoint-observation weighting remained conditional on the existing predictions and estimated weights and did not include refitting uncertainty for either model.

## Results

### Analytic samples

The HRS discovery data contained 53,721 at-risk windows. The subsequent lung-disease outcome was observed in 47,720 windows, including 881 events. PEF and outcome data were both available in 19,499 windows with 338 events; the principal systemic model included 18,510 windows from 9,391 participants and 320 events. The person-disjoint HRS replication risk set contained 18,748 windows from 12,446 participants and 319 events. PEF and outcome data were both available in 11,986 windows with 199 events, and the systemic model included 11,424 windows from 7,847 participants and 188 events. Most exclusions therefore arose before covariate adjustment, from physical-measure availability and outcome observation. Once PEF and outcome were available, missing systemic covariates removed 5.1% of discovery windows and 4.7% of replication windows. In ELSA, 2,670 of 2,849 eligible participants (93.7%) had at least one observed lung-disease follow-up; they contributed 8,634 person-waves and 95 first chronic lung-disease reports. A fixed wave-10 endpoint was classifiable for 1,584 participants, of whom 1,489 were observed event-free at wave 10, and 1,506 participants had an observed outcome at every follow-up wave (Fig 1 and S1 Table).

Among HRS risk windows with usable baseline PEF, 790 losses in discovery were classified as death before next-wave ascertainment, 892 as other nonresponse or unknown status, and one as withdrawal; the corresponding counts in person-disjoint replication were 572 deaths and 616 other nonresponses. Deaths were excluded rather than coded as event-free. Among windows with an observed next-wave outcome, differences between records with and without usable baseline PEF were modest in discovery (maximum absolute standardized difference, 0.17) but larger in replication (0.42), particularly for age and functional limitations (S2 Table).

Among the 2,849 baseline-eligible ELSA participants, 95 had a first report and 1,489 were observed event-free at wave 10; the remaining 1,265 had unclassifiable horizon status. The available interview-status fields contained no death-specific terminal codes, so death could not be distinguished reliably from other nonresponse in ELSA.

Participants in the systemic-model samples had a mean age of 66.7 years in HRS discovery, 67.3 years in HRS replication, and 69.2 years in ELSA. Women comprised 57.5%, 57.9%, and 57.0% of the respective samples. Current smoking was reported in 12.3% of HRS discovery windows, 12.6% of replication windows, and 8.9% of ELSA participants. The nominal lower-decile HRS threshold identified 8.3% of discovery and 8.1% of replication windows; the empirical lower decile identified 9.5% of ELSA participants (Table 1).

**Table 1:** Characteristics of the analytic samples.

Characteristic	HRS discovery	HRS person-disjoint replication	ELSA cohort analysis
Analytic units, n	18,510 windows	11,424 windows	8,634 person-waves
Unique participants, n	9,391	7,847	2,670
First lung-disease reports, n	320	188	95
Age, years	66.7 (10.2)	67.3 (10.1)	69.2 (6.9)
Women, n (%)	10,648 (57.5)	6,618 (57.9)	1,521 (57.0)
Higher/tertiary education, n (%)	4,772 (25.8)	2,863 (25.1)	455 (17.0)
Body mass index, kg/m2	28.6 (5.9)	28.5 (5.9)	28.1 (4.9)
Ever smoker, n (%)	9,788 (52.9)	6,223 (54.5)	1,671 (62.6)
Current smoker, n (%)	2,269 (12.3)	1,434 (12.6)	237 (8.9)
Self-rated health (1–5)	2.7 (1.0)	2.7 (1.0)	2.6 (1.0)
CES-D score (0–8)	1.3 (1.9)	1.3 (1.9)	1.0 (1.6)
Low PEF (cohort-specific definition), n (%)	1,531 (8.3)	930 (8.1)	253 (9.5)

Values are mean (SD) or n (%). HRS columns describe complete-record observation windows used by the systemic-adjusted models; participants can contribute more than one eligible window. ELSA baseline characteristics are person-level among participants with at least one observed follow-up, while its model uses repeated person-waves. Higher education is >=16 years in HRS and the harmonized tertiary category in ELSA. Low PEF is a residual z score below −1.2816 in HRS and below 77% predicted in ELSA.

### HRS discovery and person-disjoint replication

In the HRS discovery sample, each 1-SD lower adjusted PEF was associated with higher odds of a first lung-disease report in the systemic model (OR 1.72, 95% CI 1.49–1.98, P = 6.72 × 10^−14^). The OR comparing low- versus higher-PEF windows was 3.36 (95% CI 2.54–4.45, P = 3.10 × 10^−17^).

The person-disjoint replication sample gave an OR of 1.88 per 1-SD lower adjusted PEF (95% CI 1.58–2.24, P = 2.14 × 10^−12^) and an OR of 3.14 for low versus higher PEF (95% CI 2.19–4.49, P = 3.97 × 10^−10^). With physical-measure weights, the corresponding ORs were 1.77 (95% CI 1.41–2.22, P = 7.00 × 10^−7^) and 2.83 (95% CI 1.82–4.40, P = 4.08 × 10^−6^). Continuous estimates were similar across the two replication windows. The exploratory post-event analysis was inconclusive in replication (beta −0.122, 95% CI −0.251 to 0.007, P = 0.064); endpoint-PEF-observation weighting produced beta −0.118 (95% CI −0.249 to 0.014, P = 0.079). S2 Table reports the prespecified sensitivity analyses.

### ELSA cohort analysis

In ELSA, each 10 percentage-points lower percent-predicted PEF was associated with higher odds of a first observed chronic lung-disease report in the systemic model (OR 1.31, 95% CI 1.17–1.48, P = 7.02 × 10^−6^). Participants in the empirical lower PEF decile had an OR of 3.77 (95% CI 2.36–6.01, P = 2.66 × 10^−8^). Survey-plus-response weighting gave an OR of 1.36 per 10 percentage-points lower PEF (95% CI 1.21–1.53, P = 5.23 × 10^−7^) and an OR of 4.38 for low PEF (95% CI 2.67–7.19, P = 5.02 × 10^−9^).

Results were also directionally consistent for the secondary combined respiratory-disease outcome, which included 185 first reports. The systemic OR was 1.25 per 10 percentage-points lower PEF (95% CI 1.15–1.37, P = 5.74 × 10^−7^), and the lower-decile OR was 3.28 (95% CI 2.25–4.77, P = 6.13 × 10^−10^). HRS and ELSA estimates are shown separately because their exposure definitions, eligibility criteria, and follow-up structures differed (Fig 2a and Table 2).

**Table 2:** Association of baseline PEF with subsequent first observed self-report of recognized disease.

Cohort	Evidence layer	Outcome	Contrast	Observations	People	Events	OR (95% CI)	P
HRS	Discovery	First observed lung-disease report	Per 1-SD lower PEF	18,510	9,391	320	1.72 (1.49-1.98)	6.72e-14
HRS	Discovery	First observed lung-disease report	Nominal lower-decile threshold vs higher PEF	18,510	9,391	320	3.36 (2.54-4.45)	3.10e-17
HRS	Person-disjoint replication	First observed lung-disease report	Per 1-SD lower PEF	11,424	7,847	188	1.88 (1.58-2.24)	2.14e-12
HRS	Person-disjoint replication	First observed lung-disease report	Nominal lower-decile threshold vs higher PEF	11,424	7,847	188	3.14 (2.19-4.49)	3.97e-10
HRS	Physical-measure weighted sensitivity	First observed lung-disease report	Nominal lower-decile threshold vs higher PEF	10,987	7,593	181	2.83 (1.82-4.40)	4.08e-06
HRS	Non-respiratory comparator	Incident stroke	Per 1-SD lower PEF	11,573	7,953	203	1.08 (0.93-1.25)	0.305
ELSA	Cohort analysis	First observed chronic lung-disease report	Per 10%-predicted lower PEF	8,634	2,670	95	1.31 (1.17-1.48)	7.02e-06
ELSA	Cohort analysis	First observed chronic lung-disease report	Empirical lower decile vs higher PEF	8,634	2,670	95	3.77 (2.36-6.01)	2.66e-08
ELSA	Survey + response-weighted sensitivity	First observed chronic lung-disease report	Empirical lower decile vs higher PEF	8,396	2,599	90	4.38 (2.67-7.19)	5.02e-09
ELSA	Adjustment for separate FEV1 and FVC indices	First observed chronic lung-disease report	Per 10%-predicted lower PEF	8,634	2,670	95	1.04 (0.90-1.20)	0.621
ELSA	Non-respiratory comparator	Incident stroke	Per 10%-predicted lower PEF	8,352	2,579	125	1.08 (0.99-1.17)	0.072
ELSA	Non-respiratory comparator	Incident stroke	Empirical lower decile vs higher PEF	8,352	2,579	125	1.16 (0.68-1.97)	0.594

All estimates are odds ratios with participant-cluster robust 95% confidence intervals. Systemic adjustment includes demographics, smoking, adiposity, comorbidity/general health, depressive symptoms, grip strength, and functional limitations, harmonized as closely as the cohorts permit. HRS continuous effects are oriented per 1-SD lower residualized PEF; ELSA continuous effects are per 10 percentage-points lower percent-predicted PEF. HRS low PEF uses a prespecified nominal lower-decile z-score threshold; ELSA low PEF uses an empirical lower decile. The ELSA spirometric-volume-adjusted model additionally includes separate GLI FEV1 and FVC deficit z scores; no FEV1/FVC ratio was analysed. Incident stroke is an imperfect non-respiratory comparator, not evidence of respiratory specificity.

**Fig 2:**
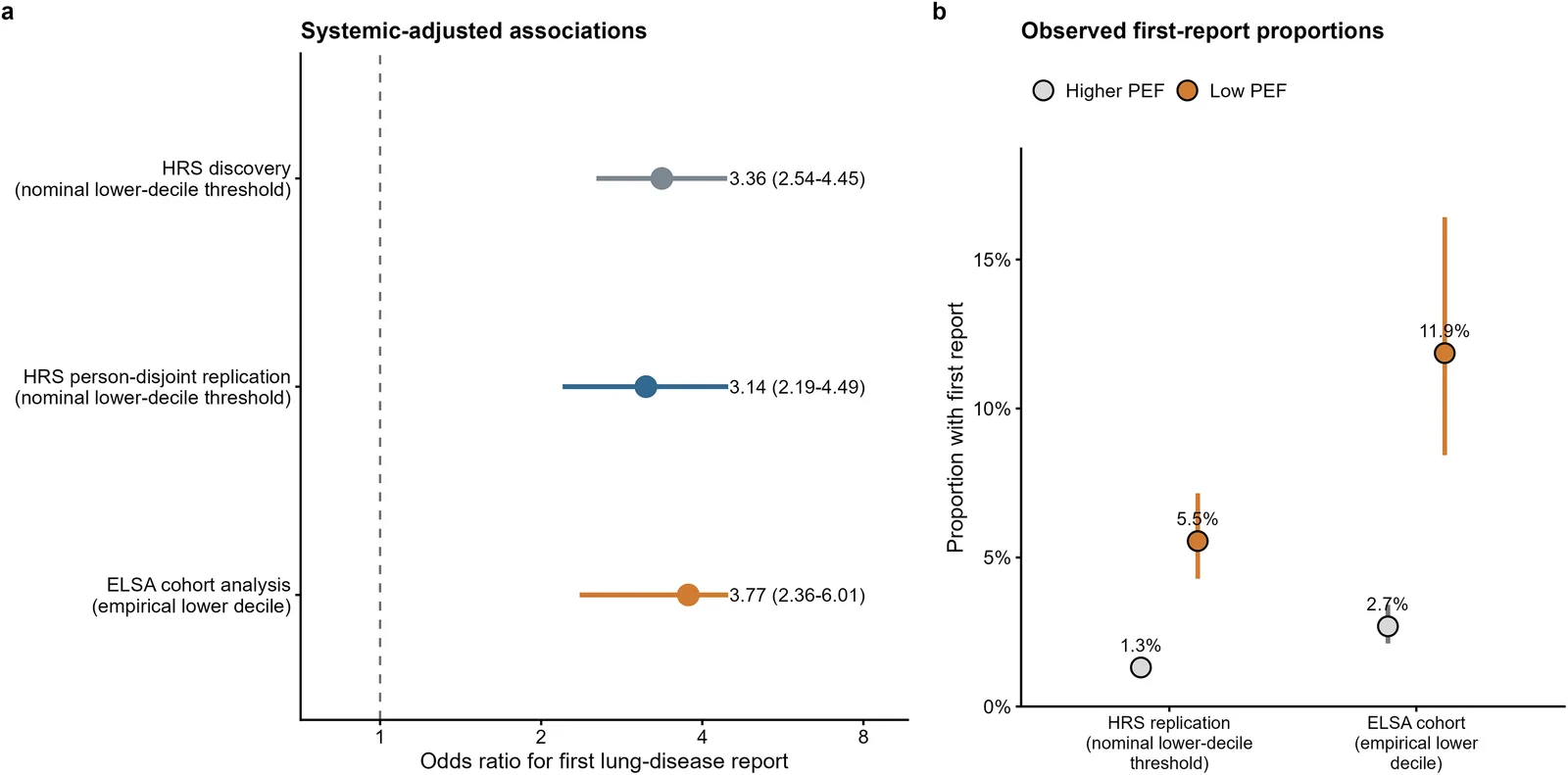
Cohort-specific associations of low peak expiratory flow with a subsequent first observed self-report of recognized lung disease. a, Systemic-adjusted odds ratios comparing cohort-specific low with higher PEF in HRS discovery windows (18,510 observation windows; 320 first reports), person-disjoint HRS replication windows (11,424 observation windows; 188 first reports), and the ELSA cohort analysis (2,670 participants; 95 first reports). HRS used a prespecified residual z-score threshold below −1.2816, corresponding to a nominal lower decile; ELSA used the empirical lower decile of percent-predicted PEF (<77% predicted). Points and horizontal lines show cohort-specific odds ratios and participant-cluster robust 95% confidence intervals; no pooled estimate is presented. b, Crude proportions with a first report among low- versus higher-PEF groups. HRS replication measured next-wave reporting, whereas ELSA accumulated first reports across waves 7-10. The proportions and operational metrics are therefore descriptive within cohorts and are not directly comparable across a common prediction horizon. Points and vertical lines show proportions and Wilson 95% confidence intervals. PEF, peak expiratory flow; HRS, Health and Retirement Study; ELSA, English Longitudinal Study of Ageing.

All six tests within each of the two respiratory-outcome families remained supported after Benjamini-Hochberg correction. The fully adjusted and weighted stroke-comparator tests did not; the interpretation of the respiratory findings did not depend on q-value thresholding.

### Absolute risk and operational performance

The relative associations corresponded to clear but clinically limited absolute-risk separation. In the broader HRS replication set with observed low-PEF status and outcome (11,986 next-wave windows; 199 first reports), 55 of 991 low-PEF windows had a first report, a crude risk of 5.5%, compared with 144 of 10,995 higher-PEF windows, a risk of 1.3%. This operational set was larger than the 11,424-window systemic complete-record model. The HRS nominal-threshold rule had 27.6% sensitivity, 92.1% specificity, 5.5% PPV, and 98.7% NPV for the next-wave outcome.

In ELSA, 30 of 253 participants with low PEF had a first chronic lung-disease report through wave 10 (11.9%), compared with 65 of 2,417 participants with higher PEF (2.7%). Sensitivity was 31.6%, specificity 91.3%, PPV 11.9%, and NPV 97.3%. Thus, low PEF marked a group with higher subsequent recognition risk but missed most later reports. Because ELSA accumulated reports across waves 7–10 whereas HRS assessed a single next-wave interval, the numerical similarity of these metrics does not represent performance over a common horizon (Fig 2b).

### Non-respiratory comparator and spirometric-volume benchmarking

Associations with incident stroke were smaller than those with lung-disease reporting. In HRS replication, each 1-SD lower adjusted PEF had an OR of 1.08 for incident stroke (95% CI 0.93–1.25, P = 0.305). In ELSA, the systemic OR was 1.08 per 10 percentage-points lower PEF (95% CI 0.99–1.17, P = 0.072), and the empirical lower-decile OR was 1.16 (95% CI 0.68–1.97, P = 0.594). Stroke shares determinants with PEF and lung-disease reporting, including smoking, frailty, cardiovascular health, and healthcare contact. These results neither establish respiratory specificity nor exclude non-specific pathways.

After separate FEV1 and FVC deficit z scores were included jointly, the continuous PEF estimate was close to unity in the ELSA systemic association model (OR 1.04 per 10 percentage-points lower PEF, 95% CI 0.90–1.20, P = 0.621); the primary low-dimensional estimate was OR 1.04 (95% CI 0.91–1.19, P = 0.548).

In the fixed-horizon evaluation set, five-repeat cross-validated AUC was 0.650 with core covariates, 0.684 after adding continuous PEF, 0.745 after adding FEV1 and FVC, and 0.739 after adding PEF to both volume indices. A separate 500-replicate full-pipeline bootstrap sensitivity reran one newly allocated five-fold cycle per replicate. Its one-cycle point estimate for adding PEF beyond FEV1 and FVC was a delta AUC of −0.0108, with a 95% percentile interval of −0.0177 to 0.0056; intervals for average precision, Brier score, and log loss also spanned zero (S1 Table). Thus, no clear evidence of incremental performance was observed in this post-hoc analysis. Without FEV1 and FVC, PEF increased average precision from 0.127 to 0.218 and reduced Brier score from 0.05514 to 0.05274 and log loss from 0.21910 to 0.21041; adding FEV1 and FVC produced larger overall gains. Across the four primary models, observed risk was 6.0%, mean predicted risk was 4.61%−4.65%, calibration-in-the-large was 0.287–0.300, and calibration slopes were 0.839–0.932. The similar calibration offset across models indicates that it was not specific to adding PEF.

Endpoint-observation weights ranged from 0.695 to 2.598 after truncation and normalization, with an effective sample size of 1,419; the largest absolute baseline standardized difference fell from 0.239 to 0.030. Weighted AUC was 0.748 with FEV1 and FVC and 0.742 after PEF was added, while changes in average precision, Brier score, and log loss remained small or imprecise. Weighted observed risk was 7.29% compared with mean predicted risks of 4.97%−5.06%, and calibration-in-the-large increased to 0.424–0.437. The strict all-waves-observed analysis (n = 1,506; 95 events) gave the same ranking and prediction-error pattern and is reported separately in S1 Table. Systemic sensitivity models also gave the same qualitative interpretation, although they generated fitted-probability warnings in 5 of 25 folds.

## Discussion

Lower PEF was associated with the subsequent first observed self-report of lung disease in HRS discovery, HRS person-disjoint replication, and ELSA. Odds ratios for the HRS nominal-threshold and ELSA empirical lower-decile contrasts were approximately three to four, and weighting gave similar point estimates. Yet the cohort-specific rules identified only 27.6% of next-wave reports in HRS and 31.6% of reports accumulated through wave 10 in ELSA; these percentages are not directly comparable. In the exploratory fixed out-of-fold predictions, adding PEF to covariates improved average precision and prediction error, whereas the full-pipeline analysis found no clear evidence of incremental performance after FEV1 and FVC. Together, these results place low PEF closer to a risk-enrichment signal than to a stand-alone screening or diagnostic test.

The temporal association agrees with earlier work in older populations. Low PEF has predicted disability, healthcare use and mortality [10,11], and Chinese longitudinal studies have linked low PEF or PEF-related trajectories with chronic lung disease, incident chronic disease and lung-function decline [22–25]. The present findings add prespecified person-disjoint HRS replication, assessment in a second national cohort and a direct comparison with other lung-function indices. They do not imply that PEF is a new disease-specific biomarker: its relation to later reporting may reflect respiratory impairment, general vulnerability, effort during testing and the process by which disease becomes recognized.

The outcome is best understood as recognition rather than onset. Airflow impairment can exist for years before a clinical label, and recorded diagnoses may disagree with post-bronchodilator spirometry [1–4]. Conversely, targeted assessment can bring previously undiagnosed disease into care [5–8]. In ELSA, participants could consent to having nurse-assessment results sent to their general practitioner. Baseline spirometry could therefore have increased subsequent clinical attention for some participants, so the ELSA association may partly reflect ascertainment or diagnostic attention triggered by the baseline assessment. Because the GP-release indicator was unavailable, the contribution of this feedback pathway could not be separated from latent disease or delayed recognition. The results locate a period of increased reporting risk after low PEF; they do not date biological onset.

The absolute-risk results limit immediate clinical use. Most participants with low PEF did not report lung disease during follow-up, and most subsequent reports occurred above the low-PEF threshold. The high NPVs chiefly reflect the low observed-report frequency. This pattern resembles COPD case-finding studies in which a single flow measure provides some enrichment but combined approaches perform better [15–19]. The distinction from population screening is important because the recommendation against COPD screening applies to asymptomatic adults, not to targeted assessment prompted by symptoms or risk features [9]. Unexpectedly low PEF may justify review of symptoms, smoking and occupational exposures, followed by quality-assured spirometry when indicated. It should not be used to assign a COPD or chronic lung-disease diagnosis, for which appropriate spirometric acquisition and interpretation remain essential [20,35,36].

The ELSA benchmarking explains why the same PEF signal can be associated with future reporting yet add little once other spirometric indices are known. PEF improved average precision and overall prediction error over core covariates, whereas FEV1 and FVC produced larger gains. No clear evidence of incremental performance after those indices was observed in this post-hoc analysis; full-pipeline intervals for all four incremental metrics spanned zero. This comparison was made within one pre-bronchodilator nurse protocol and used separately released FEV1 and FVC maxima, not a valid FEV1/FVC ratio. It therefore shows overlapping information among the recorded indices, not equivalence between peak-flow measurement and diagnostic spirometry.

The fixed-horizon models also underestimated absolute risk. All four models predicted mean risks near 4.6% when observed risk was 6.0%, and endpoint-observation weighting increased rather than resolved the calibration offset. Calibration can fail even when discrimination remains acceptable [34]. Here, the fixed-horizon construction retained all observed cases but required wave-10 ascertainment for event-free participants, so the scored set was selected partly through the observed outcome process; the small event count and lack of a separate recalibration sample provide further likely explanations. Weighting changed the contribution of observed participants but did not recalibrate predictions. The benchmarking results are therefore useful for comparing information gain, not for individual risk estimation.

Several features reduce common sources of optimistic interpretation. Repeat HRS contributors were separated between discovery and replication, participant identity defined the cross-validation folds, and preprocessing was learned within each training fold. Absolute risks and case-finding metrics were reported alongside ORs. Weighting addressed measured components of selection into baseline measurement or endpoint observation, within the limits of each weight. Incident stroke provided an imperfect non-respiratory comparison; its smaller estimates do not establish respiratory specificity because stroke and lung-disease reporting share determinants and differ in ascertainment.

The study also has important limitations. Lung disease was self-reported and could not be consistently separated into COPD, asthma, interstitial disease, or other diagnoses. Medical records and post-bronchodilator spirometry were unavailable for outcome adjudication. HRS physical measures were collected in a half-sample, and exposure and outcome availability accounted for most exclusions: usable PEF was present in 19,499 of 47,720 discovery windows and 11,986 of 16,167 replication windows with an observed next-wave outcome. PEF-available records were somewhat younger and less functionally impaired, particularly in replication (maximum absolute standardized difference, 0.42). Once PEF and outcome were available, systemic complete-record models retained 94.9% and 95.3% of windows, respectively. The physical-measure respondent weights address the baseline examination design and measured participation, not longitudinal outcome nonresponse, death, or unmeasured selection. The HRS operational analysis used the larger exposure-outcome sample rather than the systemic-model sample. ELSA was restricted to White participants under the available prediction equation and cohort definition, which limits generalizability.

The benchmarking analysis included only 95 ELSA events and was post hoc. Its high-dimensional systemic models had about three events per parameter and generated extreme fitted-probability warnings in 5 of 25 folds. The fixed-horizon estimand applies only to participants classifiable under the study observation rule, not all baseline-eligible participants: 1,265 of 2,849 eligible participants had unclassifiable horizon status. The available ELSA interview-status fields did not reliably distinguish death from other nonresponse. Endpoint-observation weighting is not sequential censoring adjustment and cannot reconstruct intermittent nonresponse, death, withdrawal, and disease recognition. The new full-pipeline bootstrap incorporated participant resampling, fold allocation, preprocessing, refitting, prediction, and evaluation, but each bootstrap used one five-fold partition and did not remedy fixed-horizon selection. Endpoint-weighted intervals remained conditional on the existing predictions and estimated weights. Finally, cohort-specific PEF scales, eligibility criteria, disease questions, and follow-up horizons precluded formal pooling. The inconclusive HRS post-event analysis provides no support for a bidirectional dynamic cascade.

## Conclusions

Lower PEF was associated with a subsequent first observed self-report of recognized lung disease in HRS discovery, person-disjoint replication, and ELSA, but cohort-specific low-PEF rules detected fewer than one-third of reports. In the exploratory fixed out-of-fold predictions, adding PEF to covariates improved average precision and prediction error; the full-pipeline analysis found no clear evidence of incremental performance after FEV1 and FVC. Low PEF may help enrich a case-finding pathway, but its value should be tested prospectively against verified diagnoses and cannot be inferred as a substitute for spirometry.

## Supporting information

S1 Table ELSA spirometric-volume benchmarking, fixed-horizon disposition, full-pipeline uncertainty, and selection-sensitivity analyses. (DOCX)

S2 Table HRS participant flow, missingness, death and other nonresponse, selection comparison, and prespecified replication sensitivities. (DOCX)

S1 Code Reproducible analysis code and documentation. ZIP archive containing the author-generated R and Python scripts used for the reported analyses, a README describing run order and restricted-data access requirements, software and package-version information, and an open-source licence. The archive contains no HRS or ELSA participant-level data.
(ZIP)
