# Supplementary material for: Low peak expiratory flow and subsequent first self-report of recognized lung disease: Person-disjoint replication and spirometric benchmarking in HRS and ELSA
Source: PLoS One. 2026 Oct 12;21(10):e0360668. doi: 10.1371/journal.pone.0360668 (PMC13657285; doi:10.1371/journal.pone.0360668)
Supplement: Supplementary file 1 [file pone.0360668.s001.docx]

S1 Table. Exploratory same-protocol spirometric-volume benchmarking of peak expiratory flow in ELSA

# Panel A. Analysis sample and fixed-horizon disposition

| **Sample stage** | **Participants** | **First-report events** |
| --- | --- | --- |
| Eligible wave-6 risk set | 2,849 | - |
| At least one observed follow-up | 2,670 | 95 |
| First-report component of fixed-horizon set | 95 | 95 |
| Observed event-free wave-10 component | 1,489 | 0 |
| Fixed wave-10 evaluation set | 1,584 | 95 |
| Horizon status unclassifiable under the observation rule | 1,265 | - |
| All follow-up waves observed (strict sensitivity) | 1,506 | 95 |

# Panel B. PEF associations with first observed self-report of recognized chronic lung disease

| **Adjustment set** | **Reference model** | **Participants / person-waves** | **Events per parameter** | **OR per 10-percentage-point lower predicted PEF (95% CI)** | **Cluster-robust P** |
| --- | --- | --- | --- | --- | --- |
| Primary | Covariates only | 2,670 / 8,634 | 6.33 | 1.31 (1.17 to 1.47) | 2.19e-06 |
| Primary | Covariates + separate FEV1 and FVC indices | 2,670 / 8,634 | 5.59 | 1.04 (0.91 to 1.19) | 0.548 |
| Systemic (secondary) | Covariates only | 2,670 / 8,634 | 3.17 | 1.31 (1.17 to 1.48) | 7.02e-06 |
| Systemic (secondary) | Covariates + separate FEV1 and FVC indices | 2,670 / 8,634 | 2.97 | 1.04 (0.90 to 1.20) | 0.621 |

# Panel C. Repeated-cross-validation performance in the fixed wave-10 evaluation set

| **Model** | **ROC AUC (95% CI)** | **Average precision (95% CI)** | **Brier score (95% CI)** | **Log loss (95% CI)** | **Calibration-in-the-large** | **Calibration slope** |
| --- | --- | --- | --- | --- | --- | --- |
| Primary covariates | 0.650 (0.592 to 0.711) | 0.127 (0.090 to 0.186) | 0.05514 (0.04525 to 0.06539) | 0.21910 (0.18453 to 0.25439) | 0.287 | 0.839 |
| Primary covariates + continuous PEF | 0.684 (0.621 to 0.747) | 0.218 (0.146 to 0.308) | 0.05274 (0.04309 to 0.06249) | 0.21041 (0.17594 to 0.24517) | 0.293 | 0.911 |
| Primary covariates + separate FEV1 and FVC indices | 0.745 (0.688 to 0.801) | 0.252 (0.172 to 0.345) | 0.05127 (0.04183 to 0.06077) | 0.20016 (0.16664 to 0.23337) | 0.300 | 0.932 |
| Primary covariates + separate FEV1 and FVC indices + continuous PEF | 0.739 (0.683 to 0.796) | 0.255 (0.173 to 0.349) | 0.05122 (0.04181 to 0.06068) | 0.20089 (0.16677 to 0.23414) | 0.300 | 0.921 |
| Systemic covariates | 0.691 (0.633 to 0.747) | 0.137 (0.099 to 0.194) | 0.05542 (0.04536 to 0.06572) | 0.22669 (0.18807 to 0.26850) | 0.305 | 0.554 |
| Systemic covariates + continuous PEF | 0.710 (0.652 to 0.768) | 0.188 (0.132 to 0.270) | 0.05320 (0.04352 to 0.06314) | 0.21813 (0.18034 to 0.25730) | 0.313 | 0.674 |
| Systemic covariates + separate FEV1 and FVC indices | 0.768 (0.715 to 0.817) | 0.225 (0.159 to 0.312) | 0.05198 (0.04253 to 0.06154) | 0.20873 (0.17062 to 0.24938) | 0.312 | 0.723 |
| Systemic covariates + separate FEV1 and FVC indices + continuous PEF | 0.762 (0.708 to 0.813) | 0.226 (0.157 to 0.313) | 0.05194 (0.04245 to 0.06155) | 0.20953 (0.17084 to 0.25045) | 0.313 | 0.716 |

# Panel D. Conditional paired incremental performance from fixed out-of-fold predictions

| **Comparison** | **Delta ROC AUC (95% CI)** | **Delta average precision (95% CI)** | **Delta Brier (95% CI)** | **Delta log loss (95% CI)** |
| --- | --- | --- | --- | --- |
| Primary: add continuous PEF to covariates | 0.03448 (−0.00666 to 0.07612) | 0.09108 (0.04041 to 0.14478) | −0.002402 (−0.003853 to −0.001083) | −0.008693 (−0.016101 to −0.001722) |
| Primary: add separate FEV1 and FVC indices to covariates | 0.09462 (0.04473 to 0.14136) | 0.12474 (0.06127 to 0.19685) | −0.003869 (−0.006265 to −0.001511) | −0.018942 (−0.029140 to −0.008677) |
| Primary: add continuous PEF beyond separate FEV1 and FVC indices | −0.00558 (−0.01012 to −0.00140) | 0.00310 (−0.00505 to 0.01409) | −0.000060 (−0.000226 to 0.000097) | 0.000737 (−0.000193 to 0.001696) |
| Systemic: add continuous PEF to covariates | 0.01996 (−0.01468 to 0.05535) | 0.05086 (0.01740 to 0.09417) | −0.002220 (−0.003719 to −0.000783) | −0.008555 (−0.016344 to −0.001312) |
| Systemic: add separate FEV1 and FVC indices to covariates | 0.07713 (0.03681 to 0.11924) | 0.08810 (0.03686 to 0.15026) | −0.003442 (−0.005999 to −0.000884) | −0.017960 (−0.028519 to −0.007141) |
| Systemic: add continuous PEF beyond separate FEV1 and FVC indices | −0.00599 (−0.01034 to −0.00209) | 0.00018 (−0.00446 to 0.00922) | −0.000044 (−0.000194 to 0.000092) | 0.000802 (−0.000065 to 0.001695) |

# Panel E. Strict complete-horizon sensitivity analysis

| **Model** | **ROC AUC** | **Average precision** | **Brier score** | **Log loss** | **Calibration-in-the-large** | **Calibration slope** |
| --- | --- | --- | --- | --- | --- | --- |
| Primary covariates | 0.651 | 0.133 | 0.05784 | 0.22805 | 0.341 | 0.838 |
| Primary covariates + continuous PEF | 0.686 | 0.230 | 0.05522 | 0.21868 | 0.352 | 0.920 |
| Primary covariates + separate FEV1 and FVC indices | 0.745 | 0.258 | 0.05377 | 0.20836 | 0.353 | 0.926 |
| Primary covariates + separate FEV1 and FVC indices + continuous PEF | 0.739 | 0.261 | 0.05370 | 0.20911 | 0.353 | 0.916 |
| Systemic covariates | 0.691 | 0.142 | 0.05816 | 0.23627 | 0.357 | 0.547 |
| Systemic covariates + continuous PEF | 0.712 | 0.197 | 0.05571 | 0.22694 | 0.372 | 0.676 |
| Systemic covariates + separate FEV1 and FVC indices | 0.768 | 0.232 | 0.05448 | 0.21747 | 0.364 | 0.718 |
| Systemic covariates + separate FEV1 and FVC indices + continuous PEF | 0.762 | 0.232 | 0.05443 | 0.21827 | 0.367 | 0.711 |

# Panel F. Endpoint-observation weighted selection sensitivity

| **Diagnostic** | **Value** |
| --- | --- |
| Source longitudinal cohort / fixed-horizon observed | 2,670 / 1,584 |
| Fixed-horizon events | 95 |
| Truncated normalized weight range | 0.695 to 2.598 |
| Effective sample size | 1,419 |
| Maximum absolute standardized difference, before / after weighting | 0.239 / 0.030 |

| **Model** | **ROC AUC** | **Average precision** | **Brier score** | **Log loss** | **Calibration-in-the-large** | **Calibration slope** | **Mean predicted risk** | **Weighted observed risk** |
| --- | --- | --- | --- | --- | --- | --- | --- | --- |
| Primary covariates | 0.656 | 0.164 | 0.06590 | 0.25333 | 0.426 | 0.853 | 0.0497 | 0.0729 |
| Primary covariates + continuous PEF | 0.691 | 0.265 | 0.06248 | 0.24187 | 0.425 | 0.914 | 0.0504 | 0.0729 |
| Primary covariates + separate FEV1 and FVC indices | 0.748 | 0.293 | 0.06092 | 0.23119 | 0.437 | 0.944 | 0.0506 | 0.0729 |
| Primary covariates + separate FEV1 and FVC indices + continuous PEF | 0.742 | 0.297 | 0.06081 | 0.23191 | 0.436 | 0.931 | 0.0506 | 0.0729 |

| **Paired comparison** | **Delta ROC AUC (95% CI)** | **Delta average precision (95% CI)** | **Delta Brier (95% CI)** | **Delta log loss (95% CI)** |
| --- | --- | --- | --- | --- |
| Add continuous PEF beyond separate FEV1 and FVC indices | −0.00531 (−0.00987 to −0.00135) | 0.00429 (−0.00574 to 0.01428) | −0.000107 (−0.000368 to 0.000151) | 0.000712 (−0.000406 to 0.002054) |

# Panel G. Full-pipeline participant-bootstrap sensitivity for the primary incremental comparison (one five-fold cycle per replicate)

| **Comparison** | **Metric** | **One-cycle point delta (extended minus reference)** | **Full-pipeline 95% percentile CI** | **Favorable direction** |
| --- | --- | --- | --- | --- |
| Add continuous PEF beyond separate FEV1 and FVC indices | ROC AUC | -0.01076 | -0.01771 to 0.00560 | Higher |
|  | Average precision | -0.00175 | -0.01910 to 0.02057 | Higher |
|  | Brier score | 0.000108 | -0.000767 to 0.000645 | Lower |
|  | Log loss | 0.001811 | -0.001123 to 0.003728 | Lower |

## Notes

This was an exploratory, post-hoc benchmarking analysis. PEF, FEV1, and FVC were co-collected in the pre-bronchodilator wave-6 nurse protocol. FEV1 and FVC were separate released session-maximal, GLI-standardized deficit indices; no FEV1/FVC ratio was exported, reconstructed, or analysed. The comparison therefore concerns information among co-collected indices, not a separate peak-flow device or a setting without spirometry. The primary prediction adjustment set was low-dimensional; the systemic set was an overfitting-sensitive sensitivity analysis. Learned preprocessing was estimated within each training fold, and participants did not cross folds. Association models used 8,634 person-waves from 2,670 participants with 95 events and participant-clustered robust inference.

The fixed-horizon target was performance among participants whose wave-10 status was classifiable under the study rule, not all 2,849 baseline-eligible participants. It comprised all 95 first reports plus 1,489 participants observed event-free at wave 10; 1,265 had unclassifiable horizon status. Waves 7-10 status fields contained no death-specific terminal codes in the eligible extract, so death could not be separated reliably from other nonresponse. The strict set required observed outcomes at every follow-up wave (n=1,506; 95 events). Five-fold participant-disjoint cross-validation was repeated five times for point predictions in Panels C-F. Their 2,000-resample paired intervals are conditional on those fixed predictions. Panel G is a separate full-pipeline sensitivity using a one-cycle estimator: its displayed point delta comes from one newly allocated five-fold cycle, and each of 500 participant bootstrap replicates repeated resampling, fold allocation, fold-local preprocessing, all four primary fits, out-of-fold prediction, and performance estimation in one five-fold cycle. Duplicate occurrences of an original participant remained in one fold. All replicates succeeded, so Panel G intervals incorporate sampling, fold-allocation, preprocessing, fitting, and evaluation uncertainty. Delta is extended minus reference; higher AUC and average precision and lower Brier score and log loss are favorable.

Panel F used stabilized endpoint-observation weights within 2,670 participants with observed follow-up. A baseline nuisance model predicted whether wave-10 status was classifiable; weights were truncated at the first and 99th percentiles, normalized, and applied to the averaged predictions. This is not time-dependent censoring weighting and cannot recover the 179 participants with no follow-up or reconstruct death, withdrawal, or recognition. Its intervals remain conditional on existing predictions and weights. All original cross-validation models converged; primary models were warning-free, whereas all four systemic specifications produced fitted-probability warnings in 5 of 25 fold partitions and had 2.38-2.81 training events per parameter. PEF, peak expiratory flow; FEV1, forced expiratory volume in 1 second; FVC, forced vital capacity; GLI, Global Lung Function Initiative; CI, confidence interval; OR, odds ratio.
