# Supplementary material for: Low peak expiratory flow and subsequent first self-report of recognized lung disease: Person-disjoint replication and spirometric benchmarking in HRS and ELSA
Source: PLoS One. 2026 Oct 12;21(10):e0360668. doi: 10.1371/journal.pone.0360668 (PMC13657285; doi:10.1371/journal.pone.0360668)
Supplement: Supplementary file 2 [file pone.0360668.s002.docx]

S2 Table. HRS participant flow, missingness, and prespecified replication sensitivities

# Panel A. Discovery sample flow and complete-record loss

| **Stage** | **Observation windows** | **Participants** | **First lung-disease reports** |
| --- | --- | --- | --- |
| At-risk windows | 53,721 | 24,222 | 881* |
| Next-wave outcome observed | 47,720 | 22,145 | 881 |
| Valid baseline PEF and outcome | 19,499 | 9,679 | 338 |
| Demographic model complete | 19,431 | 9,641 | 337 |
| Clinical model complete | 19,010 | 9,499 | 333 |
| Systemic model complete | 18,510 | 9,391 | 320 |

Among windows with valid PEF and an observed outcome, the systemic model retained 94.9% of windows and 94.7% of events.

# Panel B. Person-disjoint replication sample flow and complete-record loss

| **Stage** | **Observation windows** | **Participants** | **First lung-disease reports** |
| --- | --- | --- | --- |
| At-risk windows after locked person-disjoint exclusion | 18,748 | 12,446 | 319* |
| Next-wave outcome observed | 16,167 | 10,765 | 319 |
| Valid baseline PEF and outcome | 11,986 | 8,146 | 199 |
| Demographic model complete | 11,941 | 8,106 | 198 |
| Clinical model complete | 11,697 | 7,973 | 195 |
| Systemic model complete | 11,424 | 7,847 | 188 |

Among windows with valid PEF and an observed outcome, the systemic model retained 95.3% of windows and 94.5% of events.

# Panel C. Analyses locked before person-disjoint replication

| **Analysis** | **Exposure or contrast** | **Estimate (95% CI)** | **P value** | **N / events** |
| --- | --- | --- | --- | --- |
| Physical-measure weighted systemic model | Per 1-SD lower adjusted PEF | OR 1.77 (1.41 to 2.22) | 7.00e-07 | 10,987 / 181 |
| Physical-measure weighted systemic model | Low versus higher PEF | OR 2.83 (1.82 to 4.40) | 4.08e-06 | 10,987 / 181 |
| Window 9 systemic model | Per 1-SD lower adjusted PEF | OR 1.93 (1.50 to 2.48) | 3.67e-07 | 5,193 / 98 |
| Window 11 systemic model | Per 1-SD lower adjusted PEF | OR 1.79 (1.40 to 2.30) | 4.17e-06 | 6,231 / 90 |
| Descriptive between-window heterogeneity | Window 9 versus window 11 | Q=0.16; I-squared=0% | 0.693 | 2 windows |
| Stroke non-respiratory comparator after baseline-stroke exclusion | Per 1-SD lower adjusted PEF | OR 1.08 (0.93 to 1.25) | 0.305 | 11,573 / 203 |

# Panel D. Exploratory post-event PEF and endpoint-observation sensitivity

| **Analysis** | **Estimate (95% CI)** | **P value** | **N** |
| --- | --- | --- | --- |
| Discovery: first report predicting later adjusted PEF | Beta −0.149 (−0.241 to −0.057) | 0.0015 | 14,465 |
| Person-disjoint replication | Beta −0.122 (−0.251 to 0.007) | 0.064 | 8,914 |
| Replication, endpoint-PEF-observation weighted | Beta −0.118 (−0.249 to 0.014) | 0.079 | 8,914 |

Endpoint PEF was observed in 77.99% of no-event windows and 64.32% of event windows. In the endpoint-observation model, an intervening first lung-disease report predicted lower probability of endpoint PEF observation (coefficient −0.395, P=0.014). Stabilized weights were truncated to 0.850-1.511; the effective sample size was 8,781. The replication and weighted estimates were inconclusive and were not interpreted as evidence for a bidirectional dynamic cascade.

# Panel E. Death and other nonresponse before next-wave outcome ascertainment

| **Analysis** | **Baseline to outcome wave** | **Windows with usable baseline PEF** | **First report observed** | **Event-free response observed** | **Death** | **Other loss*** |
| --- | --- | --- | --- | --- | --- | --- |
| Discovery | 8 to 9 | 6,607 | 96 | 6,077 | 256 | 178 |
| Discovery | 10 to 11 | 7,675 | 120 | 7,033 | 238 | 284 |
| Discovery | 12 to 13 | 6,900 | 122 | 6,051 | 296 | 431 |
| **Discovery total** | **All three windows** | **21,182** | **338** | **19,161** | **790** | **893** |
| Person-disjoint replication | 9 to 10 | 5,952 | 101 | 5,299 | 315 | 237 |
| Person-disjoint replication | 11 to 12 | 7,222 | 98 | 6,488 | 257 | 379 |
| **Replication total** | **Both windows** | **13,174** | **199** | **11,787** | **572** | **616** |

*Other loss combines ordinary nonresponse or unknown status with withdrawal. One discovery window (wave 10 to 11) was recorded as dropped or withdrawn; all other entries in this column were ordinary nonresponse or unknown status. Deaths were excluded from the association analysis because no subsequent self-report was available; they were not coded as event-free responses.

# Panel F. Measured characteristics by baseline PEF availability among windows with an observed next-wave outcome

| **Characteristic** | **Discovery: PEF unavailable** | **Discovery: PEF available** | **SMD** | **Replication: PEF unavailable** | **Replication: PEF available** | **SMD** |
| --- | --- | --- | --- | --- | --- | --- |
| Age, years | 67.24 (10.65); n=26,508 | 66.64 (10.24); n=19,499 | -0.06 | 69.75 (11.29); n=3,467 | 67.28 (10.11); n=11,986 | -0.23 |
| Women | 16,707/28,221 (59.2%) | 11,389/19,499 (58.4%) | -0.02 | 2,516/4,181 (60.2%) | 7,031/11,986 (58.7%) | -0.03 |
| Black race | 5,157/28,221 (18.3%) | 3,415/19,499 (17.5%) | -0.02 | 808/4,181 (19.3%) | 2,023/11,986 (16.9%) | -0.06 |
| Hispanic ethnicity | 4,002/28,221 (14.2%) | 2,276/19,499 (11.7%) | -0.07 | 694/4,181 (16.6%) | 1,470/11,986 (12.3%) | -0.12 |
| Education, years | 12.59 (3.31); n=28,110 | 12.84 (3.13); n=19,431 | 0.08 | 12.15 (3.52); n=4,173 | 12.77 (3.18); n=11,941 | 0.18 |
| Body mass index, kg/m2 | 28.31 (5.89); n=27,671 | 28.62 (5.88); n=19,233 | 0.05 | 28.08 (6.03); n=4,042 | 28.55 (5.92); n=11,843 | 0.08 |
| Ever smoker | 15,283/28,027 (54.5%) | 10,251/19,379 (52.9%) | -0.03 | 2,325/4,135 (56.2%) | 6,469/11,913 (54.3%) | -0.04 |
| Current smoker | 3,779/28,021 (13.5%) | 2,392/19,377 (12.3%) | -0.03 | 635/4,134 (15.4%) | 1,503/11,911 (12.6%) | -0.08 |
| Self-rated health (1-5) | 2.82 (1.07); n=28,201 | 2.74 (1.04); n=19,490 | -0.08 | 2.93 (1.13); n=4,172 | 2.75 (1.04); n=11,978 | -0.17 |
| Hospitalization since prior interview | 6,647/27,953 (23.8%) | 4,477/19,472 (23.0%) | -0.02 | 1,038/4,140 (25.1%) | 2,684/11,964 (22.4%) | -0.06 |
| Comorbidity count | 1.68 (1.26); n=28,221 | 1.69 (1.24); n=19,499 | 0.01 | 1.70 (1.33); n=4,181 | 1.74 (1.22); n=11,986 | 0.04 |
| ADL limitations | 0.34 (0.93); n=28,107 | 0.23 (0.72); n=19,499 | -0.12 | 0.57 (1.28); n=4,172 | 0.23 (0.70); n=11,986 | -0.33 |
| IADL limitations | 0.32 (0.93); n=28,106 | 0.19 (0.62); n=19,499 | -0.17 | 0.63 (1.38); n=4,171 | 0.18 (0.61); n=11,986 | -0.42 |
| Mobility limitations | 1.06 (1.45); n=28,110 | 0.95 (1.34); n=19,499 | -0.07 | 1.28 (1.67); n=4,169 | 0.94 (1.34); n=11,986 | -0.22 |

Comparisons are descriptive and use observation windows as the analytic unit. PEF-available windows had a valid baseline PEF measurement; PEF-unavailable windows had an observed next-wave lung-disease outcome but no valid baseline PEF. Values are mean (SD) or n/N (%), with variable-specific available denominators. Standardized differences are oriented as PEF available minus PEF unavailable; no P values were calculated. The maximum absolute standardized difference was 0.17 in discovery and 0.42 in person-disjoint replication, with the larger replication differences concentrated in age and functional limitations.

## Notes

For the at-risk rows in Panels A and B, event counts refer to first reports among windows with an observed next-wave outcome; windows with an unobserved outcome could not be classified as events or non-events. HRS physical-measure availability reflects the enhanced face-to-face half-sample design as well as interview mode and setting. No exposure or outcome was imputed. Demographic, clinical, and systemic rows show complete records for the covariates required by each nested model. The wave-specific physical-measure respondent weights address the baseline half-sample design and measured differential participation in the enhanced face-to-face examination. They are not longitudinal follow-up weights, cannot restore next-wave outcomes after death or other nonresponse, and cannot remove selection related to unmeasured factors. Window-specific heterogeneity was a descriptive two-estimate check and was not used to pool effects. In association models, ORs for continuous PEF are expressed per 1-SD lower adjusted PEF. Operational low PEF in HRS was prespecified as an adjusted residual z score below -1.2816, a nominal lower-decile threshold; it was not an empirical bottom decile. Post-event beta coefficients are standardized adjusted-PEF units. PEF, peak expiratory flow; OR, odds ratio; CI, confidence interval; SMD, standardized mean difference.
